# Comparison of outcomes in patients with methicillin-susceptible *Staphylococcus aureus* (MSSA) bacteremia who are treated with β-lactam vs vancomycin empiric therapy: a retrospective cohort study

**DOI:** 10.1186/s12879-016-1564-5

**Published:** 2016-05-23

**Authors:** Davie Wong, Titus Wong, Marc Romney, Victor Leung

**Affiliations:** PGY-V Infectious Diseases Residency Training Program, University of British Columbia, Vancouver General Hospital, D 452 Heather Pavilion, 2733 Heather Street, Vancouver, BC V5Z 1 M9 Canada; Department of Pathology and Laboratory Medicine, University of British Columbia, Vancouver, BC Canada; Division of Medical Microbiology and Infection Control, Vancouver General Hospital, JPPN1, Medical Microbiology Laboratory, 899 W 12th Ave, Vancouver, BC V5Z 1 M9 Canada; Division of Medical Microbiology, St. Paul’s Hospital, Medical Microbiology Laboratory, 1081 Burrard St., Vancouver, BC V6Z 1Y6 Canada

**Keywords:** *Staphylococcus aureus*, Bacteremia, Empiric, Therapy, Beta-lactam, Vancomycin

## Abstract

**Background:**

Prior studies suggested that vancomycin may be inferior to β-lactams for the empiric treatment of methicillin-susceptible *S. aureus* (MSSA) bacteremia. We assessed whether empiric therapy with β-lactams compared to vancomycin was associated with differences in clinical outcomes in patients with MSSA bacteremia.

**Methods:**

We conducted a retrospective cohort study of adult inpatients with their first episode of MSSA bacteremia at two tertiary care hospitals in Vancouver, Canada, between 2007 and 2014. Exposure was either empiric β-lactam or vancomycin therapy. All patients received definitive treatment with cloxacillin or cefazolin. The primary outcome was 28-day mortality. Secondary outcomes were 90-day mortality, recurrent infection at 6 months, duration of bacteremia and hospital length-of-stay. Outcomes were adjusted using multivariable logistic regression.

**Results:**

Of 814 patients identified, 400 met inclusion criteria (β-lactam = 200, vancomycin = 200). Overall 28-day mortality was 8.5 % (*n*=34). There were more cases of infective endocarditis in the β-lactam than in the vancomycin group [45 (22.5 %) vs 23 (11.5 %), *p* < 0.01]. Adjusted mortality at 28 days was similar between the two groups (OR: 1.14; 95 % CI: 0.49–2.64). No differences in secondary outcomes were observed. Transition to cloxacillin or cefazolin occurred within a median of 67.8 h in the vancomycin group.

**Conclusions:**

Empiric therapy with β-lactams was not associated with differences in all-cause mortality, recurrent infection, microbiological cure or hospital length-of-stay compared to vancomycin. Vancomycin monotherapy may be appropriate for the empiric treatment of MSSA bacteremia if definitive therapy with cloxacillin or cefazolin can be initiated within 3 days.

## Background

*Staphylococcus aureus* is the leading cause of bacteremia and carries a mortality of 20–30 % in the 21st century [1, 2]. Empiric vancomycin is commonly prescribed for patients with *S. aureus* bacteremia (SAB) to cover methicillin-resistant *S. aureus* (MRSA) as up to 50–60 % of bloodstream isolates are methicillin-resistant at some centres [3–8]. However, vancomycin is inferior to semi-synthetic anti-Staphylococcal penicillins (e.g., cloxacillin) and first generation cephalosporins (e.g., cefazolin) for the definitive treatment of methicillin-susceptible *S. aureus* (MSSA) bacteremia [9–11]. Cloxacillin and cefazolin are considered the optimal agents against MSSA and both are equally efficacious in treating MSSA bacteremia [10]. Vancomycin is associated with higher rates of infection-related mortality, re-infection and bacteriologic failure compared to cloxacillin or cefazolin in the definitive treatment of MSSA bacteremia [9, 12–15]. Whether vancomycin is inferior to β-lactams for empiric therapy remains to be fully elucidated. Early studies suggested that empiric vancomycin was associated with worse outcomes compared to empiric β-lactam therapy [3, 16, 17], but more recent data did not demonstrate any differences in outcomes [15]. Although controversial, some experts recommend the addition of a β-lactam agent to vancomycin during empiric treatment to ensure optimal coverage for MSSA for patients at the highest risk of morbidity and mortality from SAB [18]. Major limitations of previous studies were failure to control for the definitive therapy prescribed when comparing empiric regimens and neglecting to specify the empiric β-lactams used [3, 15–17]. We assessed if empiric β-lactam compared to vancomycin was associated with differences in survival, recurrent infection and microbiological cure in patients with MSSA bacteremia who received definitive therapy with cloxacillin or cefazolin.

## Methods

### Patients

We performed a retrospective cohort study of adult inpatients diagnosed with their first episode of MSSA bacteremia at two tertiary care hospitals in Vancouver, Canada, between January 2007 and December 2014, inclusive. Consecutive patients were included if they had MSSA bacteremia and either cloxacillin or cefazolin was prescribed for definitive therapy (penicillin was an acceptable alternative if the isolate was proven to be susceptible). Patients were excluded if there was missing data for 28-day mortality, no empiric therapy was administered, death occurred within 24 h following diagnosis of bacteremia, or polymicrobial bacteremia. Patients were stratified based on empiric treatment with β-lactams or vancomycin. The β-lactam group received one or more of cloxacillin, cefazolin, β-lactam/β-lactamase inhibitor combination, a third generation cephalosporin or a carbapenem, with or without vancomycin. In both groups, other antimicrobials may have been prescribed during empiric and definitive therapy.

### Definitions

Bacteremia was defined as the isolation of MSSA from one or more blood culture bottles. Bacteremia identified within 72 h of hospital admission was considered community-onset, while bacteremia diagnosed after more than 72 h of hospital admission was deemed hospital-onset. Immunocompromised state was present if any of the following were described: neutropenia (≤ 1.5 × 10^9^/L), congenital immune deficiencies, or use of immunosuppressants (TNF-α inhibitors, prednisone ≥ 10 mg/day or its equivalent, cancer chemotherapy, methotrexate, cyclophosphamide, mycophenolate mofetil, calcineurin inhibitors, mTOR inhibitors, azathioprine and any other drug generally considered to significantly weaken the immune system). Definite infective endocarditis was diagnosed using the modified Duke criteria [19]. The source of bacteremia was either stated explicitly or inferred as the most likely source based on available clinical data and microbiological results. Metastatic complications included infections that occurred distant from the presumed primary source such as septic emboli, mycotic aneurysms, osteoarticular infections, and distant abscesses. Surgical source control included only procedures performed in the operating theatre. Empiric therapy began with the first dose of empiric antibiotics and ended with the start of definitive therapy. Definitive therapy began when antimicrobial susceptibilities were known and one of the following treatments was prescribed: 1) cloxacillin or cefazolin 2) discontinuation of other empiric antibiotics for patients already on cloxacillin or cefazolin empirically, or 3) continuation of empiric cloxacillin or cefazolin. Definitive therapy ended when cloxacillin or cefazolin was stopped. Time to receipt of antibiotics was measured from the time of obtaining the first positive blood culture to the time of the first dose of antibiotic. If a patient was already on antibiotics at the time of the first positive blood culture, the time to receipt of antibiotics was zero. We calculated the hours of empiric β-lactam exposure based on start and stop dates, start and stop times, and dosing frequency.

### Outcomes

Our primary outcome was 28-day all-cause in-hospital mortality. Secondary outcomes were 90-day all-cause in-hospital mortality, recurrent infection at 6 months, duration of bacteremia and hospital length-of-stay (LOS). Time to mortality was measured from the date of the first positive blood culture to the date of death. Recurrent infection occurred when MSSA bacteremia was diagnosed again following completion of a treatment course for the previous episode of MSSA bacteremia. Duration of bacteremia was the time difference between the first positive blood culture and the first negative blood culture. Patients without follow-up blood cultures were excluded from the analysis for duration of bacteremia. Hospital LOS was measured from the date of admission to the date of discharge.

### Data extraction

Patients with MSSA bacteremia were extracted from the medical microbiology laboratory information systems and medical records were reviewed. A single reviewer collected data on patient demographics and comorbidities, blood culture results and antimicrobial therapy from electronic and paper databases.

### Statistical analysis

Our predicted mortality difference between the β-lactam and vancomycin group was 15 % based on a previous study [17]. We estimated a sample size of 200 for each group to capture a 15 % difference in mortality with 80 % power at a two-tailed alpha level of 0.05. Baseline categorical variables were described as counts and percentages, and differences between groups were assessed with chi-square or Fisher’s exact tests. Continuous variables were presented as means and standard deviations, or medians and interquartile range. Differences between groups were assessed using parametric *t*-tests or non-parametric Mann–Whitney-*U* tests, as appropriate. Logistic regression methods were used to model the odds ratio of death and recurrent infection in the β-lactam compared to vancomycin group. Linear regression model was conducted for hospital LOS and duration of bacteremia. The two outcomes were log-transformed in the analysis to improve normality of the distribution of residuals. All models were adjusted for pre-specified confounding variables including age, sex, age-adjusted Charlson-comorbidity index [20], Pitt bacteremia score, infectious diseases consultation, infective endocarditis and time to receipt of empiric antibiotics. These factors have been shown to affect mortality in patients with SAB [1]. The duration of bacteremia was further adjusted for surgical source control. All analyses were performed using the SAS 9.4 software.

## Results

**Fig. 1:**
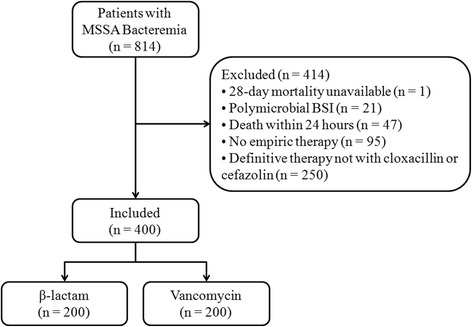
Patient enrollment process. Abbreviations: *MSSA* methicillin-susceptible *S. aureus*, *BSI* bloodstream infection

We identified 814 patients with MSSA bacteremia between January 2007 and December 2014, inclusive (Fig. 1). We excluded 414 patients primarily because 60.4 % did not receive cloxacillin or cefazolin for definitive therapy. These patients either remained on broad-spectrum antimicrobials or received vancomycin for definitive therapy due to suspected or confirmed penicillin allergy. Another 22.9 % were not started on empiric therapy. Our cohort consisted of 64.5 % males and 82.8 % of patients had community-onset bacteremia (Table 1). Infectious diseases consultation was obtained in most cases (70.5 %), but was higher in the β-lactam than in the vancomycin group (75 % vs 66 %, *p*=0.05). The most common sources of bacteremia were unknown (26 %), injection drug use (21.8 %), peripheral or central venous catheters (15.3 %), and skin and soft tissue infections (15 %). Infective endocarditis was diagnosed more frequently in the β-lactam than in the vancomycin group (22.5 % vs 11.5 %, *p* < 0.01). The prevalence of infective endocarditis was 17.0 %. The β-lactam group experienced more metastatic complications (36.5 % vs 26.5 %, *p*=0.03) and underwent surgical source control more frequently (21.5 % vs 13 %, *p*=0.02) compared to the vancomycin group.

**Table 1:** Baseline characteristics and clinical outcomes of patients with methicillin-susceptible *S. aureus* bacteremia. Patients received empiric antimicrobial therapy with either β-lactams or vancomycin

Patient characteristics	β-lactam^a^ (*n*=200)	Vancomycin^a^ (*n*=200)	*P*-value
Age^b^	53.0 ± 16.9	57.9 ± 18.4	0.01
Males	126 (63.0)	132 (66.0)	0.53
Community-onset	173 (86.5)	158 (79.0)	0.05
Hospital-onset	27 (13.5)	42 (21.0)	0.05
HIV infection	20 (10.0)	16 (8.0)	0.48
Hepatitis C infection	61 (30.5)	55 (27.5)	0.51
Immunocompromised	18 (9.0)	16 (8.0)	0.72
Alcohol or illicit drug abuse	82 (41.0)	71 (35.5)	0.26
Intravenous drug use	64 (32.0)	56 (28.0)	0.38
Charlson comorbidity index^c^	3 (1.0–6.0)	4 (1.0–7.0)	0.01
Pitt bacteremia score^c^	1 (0–2)	1 (0–2)	0.18
Infectious diseases consultation	150 (75.0)	132 (66.0)	0.05
Source of bacteremia			
Central or peripheral line	24 (12.0)	37 (18.5)	0.09
Skin and soft tissue	36 (18.0)	24 (12.0)	0.12
Intravenous drug use	48 (24.0)	39 (19.5)	0.33
Bone or joint infection	23 (11.5)	13 (6.5)	0.11
Lung	6 (3.0)	7 (3.5)	1.00
Other	19 (9.5)	20 (10.0)	1.00
Unknown	44 (22.0)	60 (30.0)	0.09
Infective endocarditis	45 (22.5)	23 (11.5)	< 0.01
Metastatic complications	73 (36.5)	53 (26.5)	0.03
Surgical source control	43 (21.5)	26 (13.0)	0.02
Empiric antimicrobials			
β-lactam	200 (100)	75 (37.5)	< 0.0001
Cloxacillin or cefazolin	138 (69.0)	10 (5.0)	< 0.0001
3^rd^ generation cephalosporin	80 (40.0)	35 (17.5)	< 0.0001
Piperacillin-tazobactam	69 (34.5)	35 (17.5)	< 0.001
Ticarcillin-clavulanic acid	3 (1.5)	1 (0.5)	0.62
Carbapenem	8 (4.0)	4 (2.0)	0.24
Vancomycin	153 (76.5)	197 (98.5)	< 0.0001
Daptomycin	2 (1.0)	1 (0.5)	1.00
Linezolid	2 (1.0)	2 (1.0)	1.00
Other^d^	82 (41.0)	97 (48.5)	0.13
Blood culture time to positivity^e^	20.2 (16.6–25.5)	18.5 (16.2–23.3)	0.02
Duration of empiric therapy^e^	55.4 (44.2–72.5)	52.1 (39.2–75.7)	0.55
Duration of definitive therapy^f^	28 (13.0–42.0)	26.5 (11.0–42.0)	0.14
Time to receipt of empiric therapy^e^	1.92 (0.1–6.9)	10.8 (1.4–24.1)	< 0.0001
Time to receipt of β-lactam^e^	2.92 (0.3–13.8)	50.5 (4.8–75.5)	< 0.0001
Time to receipt of cloxacillin or cefazolin^e^	31.0 (13.8–50.8)	67.8 (50.3–88.0)	< 0.0001
Empiric β-lactam exposure	52.3 (39.8–71.9)^e^ 60.6 ± 39.9^g^	0 (0–16.2)^e^ 9.5 ± 38.5^g^	< 0.0001
Proportional empiric β-lactam exposure	100 (100–100)^h^ 94.5 ± 46.9^i^	0 (0–24.5)^h^ 14.0 ± 44.8^i^	< 0.0001
Primary outcome			
28-day mortality	16 (8.0)	18 (9.0)	0.72
Secondary outcomes			
90-day mortality	25 (12.5)	32 (16.0)	0.32
Recurrent infection at 6 months	7 (3.5)	8 (4.0)	0.79
Duration of bacteremia^e,j^	74.4 (48.3–130)	89.7 (56.7–132)	0.20
≥ 3 days^j^	98 (53.8)	111 (60.0)	0.25
Hospital length of stay^f^	22.5 (12.5–43.0)	22 (13.0–45.0)	0.59

^a^Variables are displayed as counts and percentages in parentheses unless otherwise specified

^b^Age is represented as a mean ± standard deviation in years

^c^Variables are expressed as a median with interquartile range in parentheses

^d^Other antimicrobials used during empiric and definitive therapy included rifampin, aminoglycosides, fluoroquinolones, macrolides, trimethoprim-sulfamethoxazole, and clindamycin

^e^Variables are expressed as median hours with interquartile range in parentheses

^f^Variables are expressed as median days with interquartile range in parentheses

^g^Variables are expressed a mean ± standard deviation in hours

^h^Variables are expressed as median percentages with interquartile range in parentheses

^i^Variables are expressed a mean percentage ± standard deviation

^j^Data missing for 18 and 15 patients in the β-lactam and vancomycin group, respectively

The most common empiric antimicrobials prescribed in the β-lactam group were vancomycin (76.5 %), cloxacillin or cefazolin (69 %), 3^rd^ generation cephalosporins (40 %) and piperacillin-tazobactam (34.5 %). The use of multiple β-lactam antibiotics reflects changes made during empiric therapy. Among the subgroup of patients who received combination therapy with β-lactam plus vancomycin (153/200), cloxacillin or cefazolin (62.1 %), 3rd generation cephalosporins (47.7 %) and piperacillin-tazobactam (42.5 %) were the most common empiric β-lactams prescribed (Table 2). Cloxacillin or cefazolin (91.5 %) was the predominant empiric β-lactam used in the monotherapy subgroup. Initiation of cloxacillin or cefazolin was delayed in the combination subgroup compared to the β-lactam monotherapy subgroup (median 34.8 vs 13.0 h, *p*=0.00). The combination subgroup had a higher Pitt bacteremia score (median 1 vs 0, *p* < 0.01), received more infectious diseases consultations (78.4 % vs 63.8 %, *p*=0.05), and experienced more metastatic complications (43.1 % vs 14.9 %, *p* < 0.001) than the β-lactam monotherapy subgroup. Both the duration of bacteremia (median 84.8 vs 63.4 h, *p*=0.03) and hospital LOS (median 26 vs 15 days, *p* < 0.01) were longer in the combination subgroup.

**Table 2:** Baseline characteristics and clinical outcomes of patients with methicillin-susceptible *S. aureus* bacteremia. Patients received either empiric combination therapy with β-lactam plus vancomycin or empiric β-lactam monotherapy

Patient characteristics	β-lactam plus vancomycin^a^ (*n*=153)	β-lactam monotherapy^a^ (*n*=47)	*P*-value
Age^b^	51.3 ± 16.9	58.7 ± 16.1	< 0.01
Males	92 (60.1)	34 (72.3)	0.17
Community-onset	137 (89.5)	36 (76.6)	0.03
Hospital-onset	16 (10.5)	11 (23.4)	0.03
HIV infection	18 (11.8)	2 (4.26)	0.17
Hepatitis C infection	50 (32.7)	11 (23.4)	0.28
Immunocompromised	12 (7.84)	6 (12.8)	0.38
Alcohol or illicit drug abuse	68 (44.4)	14 (29.8)	0.09
Intravenous drug use	54 (35.3)	10 (21.3)	0.08
Charlson comorbidity index^c^	3 (1–6)	3 (1–5)	0.30
Pitt bacteremia score^c^	1 (0–2)	0 (0–1)	< 0.01
Infectious diseases consultation	120 (78.4)	30 (63.8)	0.05
Source of bacteremia			
Central or peripheral line	15 (9.80)	9 (19.1)	0.12
Skin and soft tissue	25 (16.3)	11 (23.4)	0.28
Intravenous drug use	43 (28.1)	5 (10.6)	0.02
Bone or joint infection	15 (9.80)	8 (17.0)	0.19
Lung	4 (2.61)	2 (4.26)	0.63
Other	14 (9.15)	5 (10.6)	0.78
Unknown	37 (24.2)	7 (14.9)	0.23
Infective endocarditis	40 (26.1)	5 (10.6)	0.03
Metastatic complications	66 (43.1)	7 (14.9)	< 0.001
Surgical source control	31 (20.3)	12 (25.5)	0.43
Empiric antimicrobials			
Cloxacillin or cefazolin	95 (62.1)	43 (91.5)	< 0.0001
3^rd^ generation cephalosporin	73 (47.7)	7 (14.9)	< 0.0001
Piperacillin-tazobactam	65 (42.5)	4 (8.51)	< 0.0001
Ticarcillin-clavulanic acid	2 (1.31)	1 (2.13)	0.55
Carbapenem	7 (4.58)	1 (2.13)	0.68
Daptomycin	0	2 (4.26)	*0.05*
Linezolid	1 (0.65)	1 (2.13)	0.42
Other^d^	60 (39.2)	22 (46.8)	1.00
Blood culture time to positivity^e^	20.3 (16.3–25.3)	19.8 (18.0–27)	0.36
Duration of empiric therapy^e^	55.5 (44.9–73.8)	54.3 (38.8–64.5)	0.08
Duration of definitive therapy^f^	31 (14–43)	26 (12–40)	0.18
Time to receipt of empiric therapy^e^	1.6 (0.03–6.17)	3.95 (0.58–15.5)	0.08
Time to receipt of β-lactam^e^	2.77 (0.25–13.7)	3.95 (0.58–16.6)	0.74
Time to receipt of cloxacillin or cefazolin^e^	34.8 (21.2–58.4)	13.0 (1.83–23.8)	0.00
Empiric β-lactam exposure^e^	52.3 (39.8–73.8)	51.7 (38.8–64.5)	0.42
Proportional empiric β-lactam exposure^g^	100 (86.1–100)	100 (95.2–100)	0.01
Primary outcome			
28-day mortality	14 (9.15)	2 (4.26)	0.37
Secondary outcomes			
90-day mortality	21 (13.7)	4 (8.51)	0.45
Recurrent infection at 6 months	5 (3.27)	2 (4.26)	0.67
Duration of bacteremia^e,h^	84.8 (52.5–136)	63.4 (30.5–114)	0.03
≥ 3 days^h^	81 (56.3)	17 (44.7)	0.27
Hospital length of stay^f^	26 (13–45)	15 (10–30)	< 0.01

^a^Variables are displayed as counts and percentages in parentheses unless otherwise specified

^b^Age is represented as a mean ± standard deviation in years

^c^Variables are expressed as a median with interquartile range in parentheses

^d^Other antimicrobials used during empiric and definitive therapy included rifampin, aminoglycosides, fluoroquinolones, macrolides, trimethoprim-sulfamethoxazole, and clindamycin

^e^Variables are expressed as median hours with interquartile range in parentheses

^f^Variables are expressed as median days with interquartile range in parentheses

^g^Variables are expressed as median percentages with interquartile range in parentheses

^h^Data missing for 9 patients in each subgroup

Almost half of patients in the vancomycin group received additional antimicrobials during empiric or definitive therapy (Table 1). Rifampin and aminoglycosides were added for synergy in patients with prosthetic valve infective endocarditis or prosthetic joint infections. Fluoroquinolones, macrolides, trimethoprim-sulfamethoxazole, and clindamycin were used for either treatment of non-bacteremic co-infections or prophylaxis for other medical conditions. Three patients in the vancomycin group received an incomplete dose of vancomycin and were counted as not having received it.

There was a greater delay in receipt of empiric antimicrobials in the vancomycin group compared to the β-lactam group (median 10.8 vs 1.9 h, *p* < 0.0001) (Table 1). Seventy five (37.5 %) patients in the vancomycin group were briefly exposed to β-lactams during empiric therapy. However, exposure time (median 0 vs 52.3 h, *p* < 0.0001) and proportional time of exposure of the empiric period (median 0 vs 100 %, *p* < 0.0001) were miniscule compared to the β-lactam group. There were no differences in clinical outcomes between the two groups (Table 3). The overall 28-day and 90-day mortality was 34 (8.5 %) and 57 (14.3 %) respectively. Among patients with infective endocarditis, 28-day and 90-day mortality was 4 (8.89 %) and 7 (15.6 %) in the β-lactam group and 0 and 3 (13.0 %) in the vancomycin group. In the vancomycin group, the subset of patients who received brief exposure to empiric β-lactam experienced faster clearance of bacteremia compared to those who did not have any empiric β-lactam exposure (median 78.9 vs 96.3 h, *p*=0.04). In comparison with the β-lactam group, patients in the vancomycin group who did not have any empiric β-lactam exposure had slightly higher 90-day mortality [22 (17.6 %) vs 25 (12.5 %), *p*=0.01] and longer duration of bacteremia (median 96.3 vs 74.4 h, *p*=0.03), while those who were briefly exposed to empiric β-lactams exhibited no difference in clinical outcomes.

**Table 3:** Outcome analysis comparing β-lactam versus vancomycin group. Variables were adjusted for predefined confounding variables, including age, sex, age-adjusted Charlson-comorbidity index, Pitt bacteremia score, infectious diseases consultation, infective endocarditis and time to receipt of empiric antibiotics. Duration of bacteremia was further adjusted for surgical source control

Outcomes	Crude OR (95 % CI)	*P*-value	Adjusted OR (95 % CI)	*P*-value
28-day mortality	0.88 (0.44–1.78)	0.72	1.14 (0.49–2.64)	0.76
90-day mortality	0.75 (0.43–1.32)	0.32	1.01 (0.51–2.02)	0.97
Recurrent infection at 6 months	0.87 (0.31–2.45)	0.79	1.27 (0.39–4.11)	0.69
	Ratio of Mean (95 % CI)	*P*-value	Adjusted Ratio of Mean (95 % CI)	*P*-value
Duration of bacteremia	0.92 (0.78–1.07)	0.27	0.94 (0.79–1.11)	0.44
Hospital length-of-stay	0.96 (0.81–1.14)	0.65	0.95 (0.80–1.14)	0.60

Abbreviations: *OR* odds ratio, *CI* confidence interval

## Discussion

The goal of our study was to assess if empiric β-lactams compared to vancomycin was associated with differences in outcomes in patients with MSSA bacteremia. We found no differences in all-cause mortality at 28 and 90 days, recurrent infection at 6 months, duration of bacteremia or hospital LOS between patients treated with empiric β-lactam or vancomycin therapy. However, in the vancomycin group, the subset of patients who were not exposed to any empiric β-lactams had higher 90-day mortality and longer duration of bacteremia, while those who had even minimal exposure to empiric β-lactams did not have worse outcomes. Patients in the vancomycin group were older, had more medical comorbidities, were less likely to be assessed by an infectious diseases consultant, underwent fewer source control procedures, and experienced a greater delay in receipt of empiric antibiotics compared to the β-lactam group. More cases of infective endocarditis and metastatic complications were diagnosed β-lactam group. Despite the high prevalence of MRSA at both of our institutions (25 % and 38 %), only 76.5 % of patients in the β-lactam group received vancomycin empirically as well. Perhaps the awareness of MRSA was low among some treating clinicians or patients who did not receive empiric vancomycin were judged to be at low risk for MRSA infection. Although the combination of β-lactams with vancomycin exhibits synergistic killing against MRSA, neither synergy nor antagonism was observed against MSSA in vitro [21]. Therefore, the addition of vancomycin to β-lactams would not be expected to influence microbiological cure in MSSA bacteremia.

Interestingly, the differential time delay in receipt of empiric antimicrobials was unexpected. The β-lactam group received antimicrobial therapy earlier possibly because these patients were more severely ill as reflected in their higher rate of infective endocarditis and metastatic complications, despite similar Pitt bacteremia scores between the two groups. Infectious diseases consultation may have also contributed to earlier initiation of antibiotics in the β-lactam group.

In the β-lactam group, patients who received empiric β-lactam plus vancomycin were generally sicker as indicated by their higher Pitt bacteremia score, higher rate of infective endocarditis and metastatic complications, and longer duration of bacteremia and hospital LOS, compared to those who received empiric β-lactam monotherapy. The greater severity of illness in this combination subgroup may explain the initial use of broad-spectrum β-lactams (ceftriaxone or piperacillin-tazobactam), with subsequent de-escalation to cloxacillin or cefazolin in some patients during the empiric period by the infectious diseases consultant when *S. aureus* was identified in the blood culture. De-escalation occurred within a median of 34.8 h, which follows the time to positivity of the first blood culture (median 20.3 h).

The 28-day and 90-day mortality in our study was low at 34 (8.5 %) and 57 (14.3 %) respectively, but is within the range of 3.6 to 51.7 % reported in a meta-analysis of patients with MSSA bacteremia from catheter-related infections and infective endocarditis by Cosgrove et al. [22]. Definite infective endocarditis was diagnosed in 68 (17 %) of our patients, which is similar to rates reported in previous studies [10, 14, 16, 23].

In the vancomycin group, the median time to definitive treatment with cloxacillin or cefazolin was 67.8 h, which is comparable to other studies [3, 16, 17]. In the Khatib study, a delayed clearance of bacteremia (≥ 3 days) was observed in the vancomycin group compared to the β-lactam group (57.6 % vs 37.5 %). However, there were no differences in all-cause or attributable mortality between groups. Among injection drug users with predominantly right-sided MSSA infective endocarditis, Lodise et al. demonstrated that infection-related mortality was lower with empiric β-lactam than with vancomycin monotherapy (11.4 % vs 39.3 %, *p*=0.005) [17]. Even when patients were switched from vancomycin to a semi-synthetic penicillin within a median of 3 days, infection-related mortality remained high at 40.9 %. The overall mortality in this cohort was unusually high at 22.2 % compared to a rate of 0–4 % described in a systematic review by Yung et al. [24]. In contrast, a recent study by McDanel et al. found that empiric β-lactam therapy (predominantly piperacillin-tazobactam and ceftriaxone) compared to vancomycin was not associated with differences in mortality in patients with MSSA bacteremia [15]. However, the McDanel study excluded patients who received empiric vancomcyin plus β-lactams, and clinical outcomes were evaluated independent of the antimicrobial prescribed for definitive therapy.

At institutions where MRSA prevalence is significant, vancomycin is generally accepted as an appropriate empiric antimicrobial for SAB, but due to conflicting results from prior studies, the addition of a β-lactam agent to empiric therapy remains controversial. β-lactam monotherapy may be inadequate empiric treatment if the strain is methicillin-resistant, but mortality outcomes from retrospective studies are mixed [3, 6, 8, 25]. Ultimately, the choice of empiric therapy will depend on patient factors, the prevalence of MRSA in the population, and the ability of the microbiology laboratory to rapidly differentiate MSSA from MRSA. From the perspective of antimicrobial stewardship, vancomycin monotherapy seems favourable as the reduction in usage of β-lactams may decrease the potential for drug-drug interactions and adverse effects during empiric therapy for SAB.

A particular strength of our study is the inclusion of a large proportion of patients who received optimal anti-MSSA agents (cloxacillin and cefazolin) empirically. The major limitation of previous studies was the lack of transparency regarding the empiric β-lactams prescribed [3, 17]. This is important because not all β-lactams have the same activity against MSSA. In one retrospective study, second and third generation cephalosporins and β-lactam/β-lactamase inhibitor combinations were inferior to cloxacillin and cefazolin for empiric treatment of MSSA bacteremia [26]. We did not perform subgroup analysis for different β-lactams because antimicrobials were frequently switched during empiric therapy.

Our study has several limitations. The reason for the lack of difference in the primary outcome is likely multifactorial. Because of the low event rate in both groups, our study was potentially underpowered to detect a significant difference in mortality. The lower than expected death rate may be partly due to the exclusion of patients who died within 24 h of the diagnosis of SAB and of patients who remained on broad-spectrum β-lactams. This group may have represented a sicker population and thus, we may have selected for less critically ill patients. Due to the retrospective nature of this study, baseline characteristics between the two groups were significantly different, although we did attempt to control for these differences in the multivariable model. Future studies will need to employ matching strategies to eliminate this imbalance. Although there appears to be a protective effect of even brief exposure to empiric β-lactams, caution must be exercised when interpreting this data due to the small sample size and major differences in baseline characteristics between treatment groups. Because the source of bacteremia was not identified in a significant proportion of patients in both groups, clinical outcomes may have been impacted by a lack of source control. As two different microbiology laboratories were involved in the study, differences in the detection methods of MSSA may have affected the timing of definitive therapy. Obtaining subsequent blood cultures was often delayed or sometimes not performed at all, which may have led to an overestimation of the duration of bacteremia in both groups. Data regarding adverse effects were not collected due to the inherent difficulty of establishing drug-related events in a retrospective study. We were not able to determine if patients received appropriate dosing of antibiotics because data on antibiotic doses and vancomycin trough levels were not collected. A randomized controlled trial would be needed to confirm our study findings.

## Conclusions

Empiric therapy with β-lactams was not associated with differences in all-cause mortality, recurrent infection, microbiological cure or hospital LOS compared to vancomycin in patients with MSSA bacteremia. Vancomycin monotherapy may be appropriate for the empiric treatment of MSSA bacteremia if definitive therapy with cloxacillin or cefazolin can be initiated within 3 days.

## Abbreviations

BSI: bloodstream infection
LOS: length-of-stay
MRSA: methicillin-resistant *Staphylococcus aureus*
MSSA: methicillin-susceptible *Staphylococcus aureus*
SAB: *Staphylococcus aureus* bacteremia
