# Expression of Concern to: Comparison of outcomes in patients with methicillin-susceptible *Staphylococcus aureus* (MSSA) bacteremia who are treated with β-lactam vs vancomycin empiric therapy: a retrospective cohort study 

**DOI:** 10.1186/s12879-020-05284-0

**Published:** 2020-08-05

**Authors:** Davie Wong

**Affiliations:** PGY-V Infectious Diseases Residency Training Program, University of British Columbia, Vancouver General Hospital, D 452 Heather Pavilion, 2733 Heather Street, Vancouver, BC V5Z 1 M9 Canada

The Editor is issuing this Expression of Concern to alert readers to a number of issues with respect to this article (1).

The article [1] was published with the following list of authors: Davie Wong, Titus Wong, Marc Romney and Victor Leung. After publication, it came to the attention of the Editor that there is significant text overlap with [2], although both articles present different conclusions. A subsequent investigation has found that the first author, Davie Wong, performed additional research on an increased group of patients and submitted article [1] to *BMC Infectious Diseases* without the knowledge of the colleagues listed as co-authors, while another version of the article based on fewer patient records was under consideration in *Annals of Clinical Microbiology and Antimicrobials* and subsequently published as [2]. Davie Wong takes sole responsibility for the results presented in this article [1].

Article [1] presents a comparison of beta-lactams (including cloxacillin/cefazolin, ceftriaxone, piptazo,etc.) vs vancomycin, whereas article [2] is a comparison of cloxacillin/cefazolin vs vancomycin in the empiric treatment of MSSA bacteremia. This may explain the difference in the conclusions between articles (1) and (2). The institution where this research was carried out has been unable to confirm this. Therefore the Editor advises that readers interpret the data presented with caution.

In addition, the author wishes to clarify that in article [1] in the “Methods” section under “Patients” subsection, an additional sentence should be included: “The vancomycin group may or may not have been briefly exposed to β-lactam antibiotics during empiric therapy”. This is to indicate exposure to β-lactam was permitted, whereas in article [2], the sentence “The vancomycin group was not exposed to any β-lactams until the start of definitive therapy” excludes patients who had any β-lactam exposure.

All authors agree to the publication of this Editorial Expression of Concern.
